# The Death of the Queen

**Published:** 1901-01-26

**Authors:** 


### The Hospital

A Journal of

The Medical Sciences and Hospital Administration.

Vol. XXIX.—No. 748.] Edited by Sir Henry Burdett, K.C.B., and Solomon C. Smith, M.D., M.R.C.P. [January 26, 1901.

### The Death of the Queen.

The Good Queen is dead. Mourning and distress at a loss unprecedented cover the land and absorb the thoughts of the people, and on every hand the feeling is predominant that the loss we have sustained is not a mere matter of State, not a mere turn in the machinery of the Constitution, but a loss personal and individual, as of a friend. To those who have lived their lives and done their life-work, and seen their children grow up around them and in turn go out into the world, and all within the reign of our beloved Queen, the feeling of loss at finding that they have a Queen no more is utterly indescribable. To millions of her subjects the Queen has typified the Constitution. They have known no Constitution without her. She has lived so long that everything that she has done has been so right, and every duty associated in our minds with monarchy has been so admirably and unostentatiously performed that the limitation set by the natural span of life has almost been forgotten, and it has come as a shock unutterable to find that she who has been so much more than a queen—the Empress of this mighty realm, the mother and grandmother of kings, and yet the friend of the poorest in misfortune—is now dead, never to return.

It is difficult to estimate, it is perhaps impossible at the present day to understand the influence for good which has been continuously exercised by Her Majesty throughout the whole length of her long reign.

We will not speak here of politics or of that hidden force which, emanating from the throne as the source of honour, has during all these years made for political rectitude, but we must add our small tribute of recognition to that persistent siding with the right, that consistent support of virtue against laxity, that chilling non-recognition of all that savoured of impropriety, that womanly outpouring of sympathy towards every form of distress, which have been characteristic of Her Majesty's long reign.

There are people who pretend that kings and queens are puppets, wheels within the Constitution, forced to go round their given course, personages necessary for State pageants, but without influence. They are greatly mistaken.

Firmly and carefully as the Queen held herself back throughout her long reign from intervention in political affairs, it would be the greatest mistake in the world to imagine that this was the result of any weakness of character, for in truth in all her mental characteristics the good Queen Mother whose loss we now deplore was a strong personality. In these days we take virtue for granted, we assume as natural and reasonable that vice should at least not be flaunted, and that wrong doing should be punished. But if we look back to those days, more than sixty years ago, when our now dead Queen came as a young girl to the throne of England, and had to choose for herself between the right and the wrong, and had to decide, not merely in regard to her own conduct, as to which no breath could ever be raised, but in regard to what her Court should recognise and what taboo—and that in an age when, to say the least of it, Courts were not unaccustomed to considerable latitude; and when we see how firmly she took her stand, we recognise how strong was her will even from the beginning in regard to what she held to be right. It was no weakness, but her own strong will that kept her out of politics, and it has been her own strong will which through all these long years has silently but steadily exercised an untold influence for good not only over her own vast Empire, but over that family of kings who have imbibed the traditions and the educational influences of the English Court.

To the present generation the Queen has, perhaps, chiefly been known for her human sympathy with every form of sorrow, suffering, and distress. It is only quite the older among us who can recall the pageants and the ceremonies of the early days of her reign, and of her happy married life.

Then came her great sorrow, and one may almost say that a whole generation grew up to whom the Queen was an impersonal being. But when the time came for her gradually coming out of her retirement, her people recognised in her something new, and felt arising in themselves fresh love and a renewed affection towards one who now seemed not merely the Queen or the Empress, or the Head of the State, but the much-beloved Queen-Mother, watching over her people, showing herself to them once again, sympathising with their troubles, inquiring after their misfortunes, and encouraging them in their successes. There was something touchingly human in all this; and when the great Jubilee came then it was that the nation suddenly found out how they loved their Queen. And now they have lost her, and no one shall say how great is that loss, not merely to the country as a country, but to the thousands — shall we say the millions? — of her subjects who, though perhaps they may have never seen her, have yet set her up as an ideal, and have felt towards her that kind of love, respect, and affection which is at the root of much religion.

We must, however, turn from thoughts of mourning to the practical affairs of life. The Queen is dead! God rest her soul! The King lives! God Save the King! And it is an appropriate thing, and a pleasant thing, and a thing which makes for continuity, that those very things which during the later years of her life made the Queen so beloved—her sympathy with distress, her readiness to help, her association with hospitals and village nursing, and other means of bringing succour to the sick—that these are the very things in regard to which the name of the Prince of Wales, our King, has of late years been most associated. For years past he has been known to take the keenest interest in all that concerns hospitals. Not an outside academic interest, but an active personal concern; a visitor, often an unexpected visitor, to the wards, a careful observer of hospital management, a man of great general experience of the world and of the men therein, he has turned his great influence and his high position to bear on the problem of the sick and the helpless, and has striven earnestly and successfully to benefit the hospitals and the sufferers within them. Thus we see continuity of tradition; thus for years back we have watched the Prince of Wales giving practical effect to that human feeling of sympathy towards suffering which characterised her who is now dead; thus we now see the same keynote by which the Queen-Mother touched the heart-strings of her people still sounding in the work of her successor. And thus while we mourn we look forward full of hope, with confidence that right will be maintained, and that the poor and suffering will never be neglected.

GOD SAVE THE KING!

